# Somatic Mosaicism in *PIK3CA* Variant Correlates With Stereoelectroencephalography-Derived Electrophysiology

**DOI:** 10.1212/NXG.0000000000200117

**Published:** 2023-12-22

**Authors:** H. Westley Phillips, Alissa M. D'Gama, Yilan Wang, Yasmine Chahine, Michelle Chiu, Amanda C. Swanson, Banu Ahtam, Jeffrey B. Bolton, Joseph R. Madsen, Eunjung A. Lee, Sanjay P. Prabhu, Hart G. Lidov, Joanna Papadakis, August Y. Huang, Annapurna Poduri, Scellig S. Stone, Christopher A. Walsh

**Affiliations:** From the Department of Neurosurgery (H.W.P.), Stanford School of Medicine, Palo Alto, CA; Department of Neurosurgery (H.W.P., J.R.M., J.P., S.S.S.), Boston Children's Hospital, Harvard Medical School; Broad Institute of MIT and Harvard (H.W.P., Y.W., Y.C., E.A.L., A.Y.H., C.A.W.), Cambridge; Division of Genetics and Genomics (H.W.P., Y.W., E.A.L., A.Y.H., C.A.W.), Manton Center for Orphan Disease Research; Division of Newborn Medicine (A.M.D.G., B.A.), Department of Pediatrics; Epilepsy Genetics Program (A.M.D.G., J.B.B., A.P.), Department of Neurology; Department of Pediatrics (A.M.D.G., J.B.B., E.A.L., A.Y.H., C.A.W.), Harvard Medical School, Boston Children's Hospital; Program in Biological and Biomedical Sciences (Y.W.); Department of Neurology (M.C., J.B.B., A.P., C.A.W.), Boston Children's Hospital, Harvard Medical School; Translational Neuroscience Center (A.C.S.), Boston Children's Hospital; Department of Radiology (S.P.P.), Division of Neuroradiology; Department of Pathology (H.G.L.), Division of Neuropathology, Boston Children's Hospital, Harvard Medical School; and Howard Hughes Medical Institute (C.A.W.), Boston, MA.

## Abstract

**Objectives:**

Brain-limited pathogenic somatic variants are associated with focal pediatric epilepsy, but reliance on resected brain tissue samples has limited our ability to correlate epileptiform activity with abnormal molecular pathology. We aimed to identify the pathogenic variant and map variant allele fractions (VAFs) across an abnormal region of epileptogenic brain in a patient who underwent stereoelectroencephalography (sEEG) and subsequent motor-sparing left frontal disconnection.

**Methods:**

We extracted genomic DNA from peripheral blood, brain tissue resected from peri-sEEG electrode regions, and microbulk brain tissue adherent to sEEG electrodes. Samples were mapped based on an anatomic relationship with the presumed seizure onset zone (SOZ). We performed deep panel sequencing of amplified and unamplified DNA to identify pathogenic variants with subsequent orthogonal validation.

**Results:**

We detect a pathogenic somatic *PIK3CA* variant, c.1624G>A (p.E542K), in the brain tissue samples, with VAF inversely correlated with distance from the SOZ. In addition, we identify this variant in amplified electrode-derived samples, albeit with lower VAFs.

**Discussion:**

We demonstrate regional mosaicism across epileptogenic tissue, suggesting a correlation between variant burden and SOZ. We also validate a pathogenic variant from individual amplified sEEG electrode-derived brain specimens, although further optimization of techniques is required.

## Introduction

Pathogenic brain-limited somatic variants play an important role in pediatric drug-refractory epilepsy (DRE), especially when associated with malformations of cortical development (MCD).^1-4^ However, diagnosis of these variants is often hindered by a lack of access to surgically resected brain samples. Furthermore, the need for preoperative research consent, intraoperative tissue retrieval, optimal specimen handling, and costly sequencing requires substantial infrastructure and funding.^5-8^

Stereoelectroencephalography (sEEG) involves the temporary placement of depth electrodes into multiple brain regions for invasive seizure onset zone (SOZ) recording. Recently, studies have demonstrated the ability to extract brain-derived DNA from sEEG electrodes, while using the spatial organization of an electrode array to study the anatomic relationship of somatic variants and epileptogenic foci.^9-11^

Here, we identify and map a pathogenic somatic *PIK3CA* variant across the SOZ of a 9-year-old patient with DRE secondary to a diffuse left frontal MCD.

## Methods

**Figure 1:**
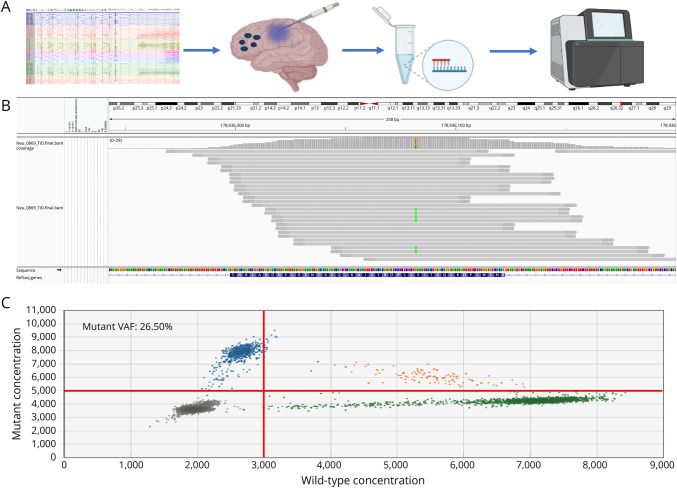
Workflow to Detect and Validate the PIK3CA E542K Variant (A) Experimental design to detect brain-limited pathogenic variants in seizure onset zones based on sEEG recordings. The genomic DNA was extracted from resected bulk-brain tissue samples, trace brain tissue adhered to sEEG electrodes, and peripheral blood; a subset of samples underwent deep-panel sequencing and variant calling. (B) The integrated genome viewer (IGV) representation of the somatic PIK3CA E542 mutation in bulk-brain sample F2b. (C) Droplet digital PCR results for bulk-brain sample F2b. Reference signal 190 copies/µl, mutant signal 68.4 copies/µl. Mutant variant allele fraction (VAF): 26.50%. Blue droplets, mutant allele; green droplets, reference allele; orange droplets, both reference and mutant DNA templates; gray droplets, empty. Of note, the VAF reported in the Table takes into account VAFs from replication and control samples.

Preoperative workup by a multidisciplinary epilepsy team was reviewed for a patient with DRE who underwent sEEG followed by a motor-sparing left frontal disconnection. Peripheral blood, resected perielectrode bulk-brain tissue, and removed sEEG electrodes were collected. Unamplified DNA was extracted using the EZ1 DNA Tissue Kit. Whole-genome amplified DNA was extracted from the microbulk brain tissue adherent to each sEEG electrode using Primary Template-directed Amplification (Figure 1A). Deep panel sequencing targeting 283 genes associated with epilepsy and related cancer pathways was performed and analyzed for a subset of samples (eTable 1, links.lww.com/NXG/A665). The candidate pathogenic variant was validated across samples using droplet-digital PCR (ddPCR) and amplicon sequencing (Figure 1, B and C). Further details on the experimental design are available in the eMethods (links.lww.com/NXG/A664).

### Study Protocols

Written informed consent was obtained by the Boston Children's Hospital (BCH) Repository Core for Neurological Diseases, a biorepository approved by the BCH Institutional Review Board.

### Data Availability

Additional data are available on request.

## Results

**Figure 2:**
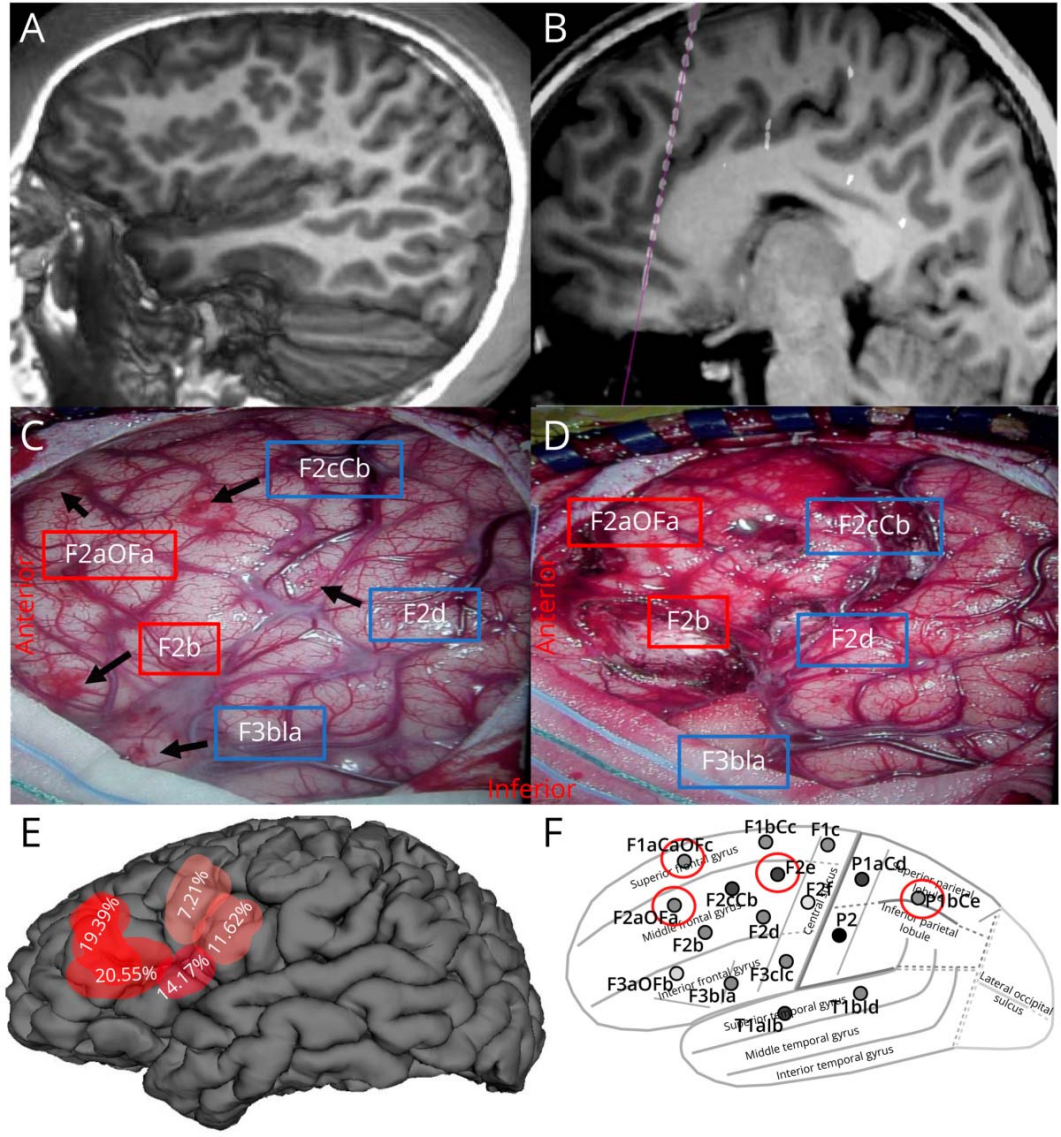
Representation of Somatic Mosaicism of Pathogenic PIK3CA E542K Variant in a 9-Year-Old Patient With Drug-Resistant Epilepsy and a Left Frontal Malformation of Cortical Development (MCD) (A) Sagittal 3D surface reconstruction of brain MRI demonstrating a diffuse left frontal malformation of cortical development on a preoperative T1-weighted image. (B) Post-sEEG electrode implantation with sagittal CT coregistration with preoperative T1-weighted MRI depicting electrode F2aOFa trajectory sampling the left anterior middle frontal gyrus to the left orbital frontal cortex. (C–D) Intraoperative microscope imaging (left is anterior) demonstrating (C) predisconnection left frontal craniotomy and (D) postmotor sparing left frontal disconnection with labeled electrode insertion sites (black arrows) indicating ictally active (red box) and inactive (blue box) regions. Of note, the electrode site corresponding to the F3aOFb was outside of the craniotomy and not shown in the image. (E) 3D reformatting of preoperative MRI with superimposed biopsy sites with color gradient (red shapes) demonstrating variant allele fraction of bulk-brain tissue derived PIK3CA E542K variant determined by deep-panel sequencing with dark red representing higher VAF relative to all samples. (F) Lateral view of sEEG implantation plan using the standard electrode nomenclature for sEEG applications naming system (SENSA) with red circles indicating electrode sites where the PIK3CA E542K VAF was detected in sEEG electrode-derived samples.

A 9-year-old left-handed girl with a history of DRE and a left-frontal lesion presented for epilepsy surgery evaluation. At the age of 4, she developed nocturnal tonic and myoclonic seizures, characterized by arousal from sleep, body stiffening, bilateral dystonic arm posturing, and vocalization. She later developed diurnal clusters of epileptic spasms marked by head drops. Her seizure frequency varied from several per day to once every few weeks. MRI demonstrated a diffuse left frontal dysplastic lesion (Figure 2, A and B). On physical examination, she had subtle right lower extremity weakness.

Her presurgical EEG demonstrated continuous left frontal interictal spikes. High-density EEG demonstrated abundant left frontal spikes with maximum amplitude in the left frontopolar, temporal, and anterior temporal regions (surface electrodes FP1, AF7, and FT9). Magnetoencephalography demonstrated left frontal high-amplitude sharp activity. Functional MRI demonstrated typical motor and right lateralizing speech representation. Her preoperative neuropsychological testing revealed borderline intellectual functioning.

The patient underwent sEEG with 17 electrodes. Electrodes were labeled using the standardized electrode nomenclature for stereoelectroencephalography applications (SENSA).^12^ Five typical tonic seizures, numerous myoclonic seizures, and several clusters of epileptic spasms were captured. Ictal onset involved the left anterior middle and inferior frontal gyrus corresponding to electrodes F2aOFa (contacts 3–8; 12-15), F2b (contacts 9–12), and F3aOFb (contacts 8–10), with early spread to the posterior frontal region, insula, and cingulate with later propagation to the left parietotemporal lobes (eFigure 1, links.lww.com/NXG/A663). Interictally, abundant spikes were apparent in the contacts involved at ictal onset. Frequent broader spikes were also seen with involvement of the left middle and inferior frontal gyri.

After sEEG, a motor-sparing left frontal disconnection was recommended. Electrodes and peripheral blood were collected. A left frontal craniotomy was performed exposing several sEEG electrode surface entry points (Figure 2C). Tissue biopsies (1 cm^3^) were taken at 5 relevant electrode sites (F2aOFa, F2b, F2cCb, F2d, F3bIa), and a left frontal disconnection was performed using microsurgical techniques (Figure 2D). Postoperatively, the patient did well and has remained seizure-free and at her neurologic baseline at 1-year follow-up. Pathologic analysis of the resected tissue revealed SMI31 positive neurons concerning for class IIa focal cortical dysplasia.

### Genetic Results

Deep panel sequencing identified a previously reported and well-characterized pathogenic gain-of-function somatic *PIK3CA* variant (chr3:178936082G>A, NM_006218.2: c.1624G>A, p.E542K) in the bulk-brain tissue samples (Figure 1). The variant was validated using ddPCR and amplicon sequencing (Figure 1C, Table). Validation using amplicon sequencing in all available samples also confirmed the presence of the variant in 4 amplified sEEG electrode-derived microbulk tissue specimens, but the variant was not detected in unamplified electrodes or blood samples. The highest variant allele fractions (VAF) were identified in the regions correlating with the ictal onset demarcated by repetitive fast activity bursts and 13 Hz rhythmic activity (Figure 2E). Samples from the F2b and F2aOFa sites, corresponding to the presumed primary SOZ, had VAFs of 20.6%–26.5% and 19.4%–21.2%, respectively, while samples derived near the less epileptogenic sites F2d, F3bIa, and F2cCb had lower VAFs of 9.5%–11.6%, 13.8%–15.5%, and 7.2%–9.7%, respectively (Table). The variant was also detected in amplified DNA derived from the F2e, F1aCaOFc, F2aOFa, and P1bCe electrodes (Figure 2F). The VAF at these sites was an order of magnitude lower (0.61%, 3.9%–5.7%, 0.38%–0.79%, and 1.04%) compared with bulk-brain samples, and the specific origin of the adherent tissue along the trajectory was unknown (eTable 2, links.lww.com/NXG/A666).

**Table:**
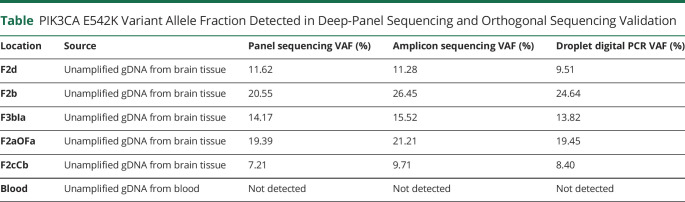
PIK3CA E542K Variant Allele Fraction Detected in Deep-Panel Sequencing and Orthogonal Sequencing Validation

Location	Source	Panel sequencing VAF (%)	Amplicon sequencing VAF (%)	Droplet digital PCR VAF (%)
F2d	Unamplified gDNA from brain tissue	11.62	11.28	9.51
F2b	Unamplified gDNA from brain tissue	20.55	26.45	24.64
F3bIa	Unamplified gDNA from brain tissue	14.17	15.52	13.82
F2aOFa	Unamplified gDNA from brain tissue	19.39	21.21	19.45
F2cCb	Unamplified gDNA from brain tissue	7.21	9.71	8.40
Blood	Unamplified gDNA from blood	Not detected	Not detected	Not detected

## Discussion

We report the ability to map a somatic PIK3CA E542K variant and its VAF gradient across a sEEG-defined SOZ. The somatic PIK3CA E542K variant is a known pathogenic gain-of-function variant annotated in the ClinVar and COSMIC databases and reported in patients with a spectrum of overgrowth syndromes, including hemimegalencephaly, providing a molecular genetic diagnosis.^13-15^

We observed higher VAFs in brain regions encompassing the SOZ defined by ictal activity, arising from the F2b and F2aOFa electrode sites, compared with other regions. The apparent regional VAF variation in this case mirrors the gradient reported by Ye et al.^10^ while higher VAFs in the SOZ suggest a dose-dependent correlation between pathogenic mutational burden and seizure activity.

Two previous studies have reported the detection of somatic variants using DNA from pooled sEEG electrodes, while recently Cherci et al. described their experience with single electrodes. Similarly, we present a comparison of single sEEG-derived microbulk tissue samples and bulk-brain specimen arising from the same brain region.^9-11^ Additional studies comparing bulk-brain tissue with electrode-derived samples are required to evaluate the accuracy of somatic variant detection and quantification.

The lower VAF observed or absence of the variant in sEEG electrode-derived DNA samples arising from implicated regions demonstrates a limitation of sequencing from this source. This is mostly likely because of heterogeneous sampling across the entire trajectory of each electrode (Figure 2B) that includes the lesion but also substantial other tissue, coupled with low DNA yield. This also decreases the anatomic resolution of our genetic analysis and limits direct correlations to the electrophysiology of sEEG in electrode-derived samples. This technique could in theory facilitate molecular analysis of brain regions outside of a region of planned surgical resection, as evidenced by the identification of the PIK3CA variant in a sample derived from P1bCe which was outside of the craniotomy (and SOZ) but demonstrated broad spiking during seizure spread (contact 15). However, in our case, the variable yield limited our ability to map anatomic-specific VAFs from electrodes alone.

Our study demonstrates a gradient of mosaicism of a pathogenic *PIK3CA* variant correlating with electrophysiologic abnormalities in an epileptogenic MCD. We demonstrate the feasibility of detecting somatic variants from sEEG electrode-derived tissue samples and highlight the necessity of comparing results from electrode and brain samples of the same region in future studies. Further technique optimization resulting in reliable and accurate sampling of sEEG-derived DNA is required.

## Appendix Authors

**Table:**

Name	Location	Contribution
H. Westley Phillips, MD	Department of Neurosurgery, Stanford School of Medicine, Palo Alto, CA; Department of Neurosurgery, Boston Children’s Hospital, Harvard Medical School; Broad Institute of MIT and Harvard, Cambridge, MA; Division of Genetics and Genomics, Manton Center for Orphan Disease Research, Boston Children’s Hospital, MA	Drafting/revision of the manuscript for content, including medical writing for content; major role in the acquisition of data; study concept or design; analysis or interpretation of data
Alissa M. D′ Gama, MD, PhD	Division of Newborn Medicine, Department of Pediatrics; Epilepsy Genetics Program, Department of Neurology; Department of Pediatrics, Harvard Medical School, Boston Children’s Hospital, MA	Drafting/revision of the manuscript for content, including medical writing for content; major role in the acquisition of data; study concept or design; analysis or interpretation of data
Yilan Wang	Broad Institute of MIT and Harvard, Cambridge; Division of Genetics and Genomics, Manton Center for Orphan Disease Research, Boston Children’s Hospital; Program in Biological and Biomedical Sciences, Harvard Medical School, Boston, MA	Drafting/revision of the manuscript for content, including medical writing for content; major role in the acquisition of data; analysis or interpretation of data
Yasmine Chahine	Broad Institute of MIT and Harvard, Cambridge, MA	Major role in the acquisition of data
Michelle Chiu, MD	Department of Neurology, Boston Children’s Hospital, Harvard Medical School, MA	Major role in the acquisition of data; analysis or interpretation of data
Amanda C. Swanson	Translational Neuroscience Center, Boston Children’s Hospital, MA	Major role in the acquisition of data
Banu Ahtam, DPhil	Division of Newborn Medicine, Department of Pediatrics, Boston Children’s Hospital, MA	Drafting/revision of the manuscript for content, including medical writing for content; major role in the acquisition of data
Jeffrey B. Bolton	Epilepsy Genetics Program, Department of Neurology; Department of Pediatrics; Department of Neurology, Boston Children’s Hospital, Harvard Medical School, MA	Drafting/revision of the manuscript for content, including medical writing for content; major role in the acquisition of data; analysis or interpretation of data
Joseph R. Madsen, MD	Department of Neurosurgery, Boston Children’s Hospital, Harvard Medical School, MA	Study concept or design; analysis or interpretation of data
Eunjung A. Lee, PhD	Broad Institute of MIT and Harvard, Cambridge; Division of Genetics and Genomics, Manton Center for Orphan Disease Research; Department of Pediatrics, Harvard Medical School, Boston Children’s Hospital, MA	Analysis or interpretation of data
Sanjay P. Prabhu, MBBS, FRCR	Department of Radiology, Division of Neuroradiology, Boston Children’s Hospital, Harvard Medical School, MA	Major role in the acquisition of data
Hart G. Lidov, MD, PhD	Department of Pathology, Division of Neuropathology, Boston Children’s Hospital, Harvard Medical School, MA	Analysis or interpretation of data
Joanna Papadakis	Department of Neurosurgery, Boston Children’s Hospital, Harvard Medical School, MA	Major role in the acquisition of data
August Y. Huang, PhD	Broad Institute of MIT and Harvard, Cambridge; Division of Genetics and Genomics, Manton Center for Orphan Disease Research; Department of Pediatrics, Harvard Medical School, Boston Children’s Hospital, MA	Drafting/revision of the manuscript for content, including medical writing for content; major role in the acquisition of data; analysis or interpretation of data
Annapurna Poduri, MD, MPH	Epilepsy Genetics Program, Department of Neurology, Boston Children’s Hospital; Department of Neurology, Boston Children’s Hospital, Harvard Medical School, MA	Drafting/revision of the manuscript for content, including medical writing for content; major role in the acquisition of data; study concept or design; analysis or interpretation of data
Scellig S. Stone, MD, PhD	Department of Neurosurgery, Boston Children’s Hospital, Harvard Medical School, Boston, MA	Drafting/revision of the manuscript for content, including medical writing for content; major role in the acquisition of data; study concept or design; analysis or interpretation of data
Christopher A. Walsh, MD, PhD	Broad Institute of MIT and Harvard, Cambridge; Division of Genetics and Genomics, Manton Center for Orphan Disease Research; Department of Pediatrics; Department of Neurology, Boston Children’s Hospital, Harvard Medical School; Howard Hughes Medical Institute, Boston, MA	Drafting/revision of the manuscript for content, including medical writing for content; major role in the acquisition of data; study concept or design; analysis or interpretation of data
